# In vitro activity of ceftazidime–avibactam and comparators against Gram-negative bacterial isolates collected in the Asia–Pacific region as part of the INFORM program (2015–2017)

**DOI:** 10.1186/s12941-020-00355-1

**Published:** 2020-04-01

**Authors:** Wen-Chien Ko, Gregory G. Stone

**Affiliations:** 1 Division of Infectious Diseases, Department of Internal Medicine, National Cheng Kung University Hospital, No. 138, Sheng Li Road, Tainan, 70403 Taiwan; 2 Pfizer Inc., 558 Eastern Point Rd, Groton, CT 06340 USA

**Keywords:** Ceftazidime–avibactam, Gram-negative, Antimicrobial susceptibility, Antimicrobial resistance, Antimicrobial surveillance, Asia–Pacific region

## Abstract

**Background:**

Antimicrobial resistance among nosocomial Gram-negative pathogens is a cause for concern in the Asia–Pacific region. The aims of this study were to measure the rates of resistance among clinical isolates collected in Asia–Pacific countries, and to determine the in vitro antimicrobial activities of ceftazidime–avibactam and comparators against these isolates.

**Methods:**

CLSI broth microdilution methodology was used to determine antimicrobial activity and EUCAST breakpoints version 9.0 were used to determine rates of susceptibility and resistance. Isolates were also screened for the genes encoding extended-spectrum β-lactamases (ESBLs) or carbapenemases (including metallo-β-lactamases [MBLs]).

**Results:**

Between 2015 and 2017, this study collected a total of 7051 Enterobacterales isolates and 2032 *Pseudomonas aeruginosa* isolates from hospitalized patients in Australia, Japan, South Korea, Malaysia, the Philippines, Taiwan, and Thailand. In the Asia–Pacific region, Enterobacterales isolates that were ESBL-positive, carbapenemase-negative (17.9%) were more frequently identified than isolates that were carbapenemase-positive, MBL-negative (0.7%) or carbapenemase-positive, MBL-positive (1.7%). Multidrug-resistant (MDR) isolates of *P. aeruginosa* were more commonly identified (23.4%) than isolates that were ESBL-positive, carbapenemase-negative (0.4%), or carbapenemase-positive, MBL-negative (0.3%), or carbapenemase-positive, MBL-positive (3.7%). More than 90% of all Enterobacterales isolates, including the ESBL-positive, carbapenemase-negative subset and the carbapenemase-positive, MBL-negative subset, were susceptible to amikacin and ceftazidime–avibactam. Among the carbapenemase-positive, MBL-positive subset of Enterobacterales, susceptibility to the majority of agents was reduced, with the exception of colistin (93.4%). Tigecycline was active against all resistant subsets of the Enterobacterales (MIC_90_, 1–4 mg/L) and among *Escherichia coli* isolates, > 90% from each resistant subset were susceptible to tigecycline. More than 99% of all *P. aeruginosa* isolates, including MDR isolates and the carbapenemase-positive, MBL-positive subset, were susceptible to colistin.

**Conclusions:**

In this study, amikacin, ceftazidime–avibactam, colistin and tigecycline appear to be potential treatment options for infections caused by Gram-negative pathogens in the Asia–Pacific region.

## Background

Antimicrobial resistance is recognized to be a healthcare issue in the Asian continent [1], where there are high levels of resistant Gram-negative organisms, such as extended-spectrum β-lactamase (ESBL)-positive Enterobacterales and multidrug-resistant (MDR) *Pseudomonas aeruginosa* [2]. Of particular concern is the presence of metallo-β-lactamase (MBL)-positive Enterobacterales and newly-emerging resistance to the polymyxins (colistin or polymyxin B) [3–5]. Recent publications have shown that further afield in the Asia–Pacific region, Australia has relatively low levels of resistant Gram-negative organisms, although carbapenem-resistant Enterobacterales and *P. aeruginosa* are emerging as health risks to the Australian public [6, 7].

Ceftazidime–avibactam, a combination of a third-generation cephalosporin and a non-β-lactam β-lactamase inhibitor, has a spectrum of activity that covers MDR Enterobacterales and *P. aeruginosa* and is able to inhibit ESBLs and *Klebsiella pneumoniae* carbapenemases (KPCs) [8]. In Australia, Thailand and Taiwan, as well as in the United States and in Europe, ceftazidime–avibactam has been approved for the treatment of complicated intra-abdominal infection (in combination with metronidazole), complicated urinary tract infection (including pyelonephritis), and hospital-acquired and ventilator-associated bacterial pneumonia [9, 10]. In Europe, ceftazidime–avibactam is also indicated for the treatment of infections due to aerobic Gram-negative organisms in adult patients with limited treatment options [10].

The aims of this study were to determine the rates of resistance among clinical isolates of Enterobacterales and *P. aeruginosa* collected in Asia–Pacific countries between 2015 and 2017, and to show the in vitro antimicrobial activities of ceftazidime–avibactam and comparators against these isolates. The countries included are Australia, Japan, South Korea, Malaysia, the Philippines, Taiwan, and Thailand. Isolates collected in 2015 are also included in an INFORM publication on the Asia–Pacific region between 2012 and 2015 [11], and isolates collected in 2015 and 2016 are also included in INFORM global publications [12, 13].

## Method

Each participating study center was required to collect a predefined number of Enterobacterales and *P. aeruginosa* isolates every year from adult and pediatric patients with specific bacterial infections. Only patient-derived, non-duplicate isolates suspected as the cause of each infection were included in the study. Isolates from external sources, such as drainage bottles or environmental samples, were excluded from the study. Demographic information was recorded for each isolate, including source of isolate and patient information, for example, age, sex and referring ward.

Isolates were identified at the local center of collection and shipped to a central laboratory (International Health Management Associates [IHMA] Inc., Schaumburg, IL, USA) for further testing. The central laboratory confirmed species identity and determined antimicrobial minimum inhibitory concentrations (MICs) using the Clinical Laboratory Standards Institute (CLSI) broth microdilution method [14]. MICs were interpreted using version 9.0 of the European Committee on Antimicrobial Susceptibility Testing (EUCAST) clinical breakpoint tables [15]. Enterobacterales and *P. aeruginosa* isolates were screened for the presence of the genes encoding ESBLs or carbapenemases (including MBLs), if they met the MIC criteria described below.

In 2015, *Escherichia coli*, *K. pneumoniae*, *Klebsiella oxytoca*, and *Proteus mirabilis* isolates with an MIC to ceftazidime or aztreonam of ≥ 2 mg/L were evaluated for ESBL activity using the CLSI phenotypic clavulanic acid combination test [14]. Isolates confirmed as having an ESBL-positive phenotype, or those that were found to be phenotypically ESBL-negative but with an MIC to ceftazidime of ≥ 16 mg/L, were screened for the genes encoding clinically-relevant β-lactamases (ESBLs: SHV, TEM, CTX-M, VEB, PER and GES; plasmid-mediated AmpC β-lactamases: ACC, ACT, CMY, DHA, FOX, MIR and MOX; serine carbapenemases: GES, KPC and OXA-48-like; and MBLs: NDM, IMP, VIM, SPM and GIM) using multiplex PCR assays, as previously described [16]. In 2016 and 2017, *E. coli*, *K. pneumoniae*, *K. oxytoca*, and *P. mirabilis* isolates with an MIC to meropenem, ceftazidime or aztreonam of ≥ 2 mg/L were screened for the presence of the above β-lactamase genes as previously described [16]. In addition, all other Enterobacterales isolates with MICs ≥ 2 mg/L to meropenem were also screened for β-lactamase genes using published multiplex PCR assays, as previously described [16]. All *P. aeruginosa* isolates with a meropenem MIC of ≥ 4 mg/L were screened for clinically-relevant β-lactamase genes (ESBLs: SHV, TEM, VEB, PER and GES; serine carbapenemases: GES, KPC and OXA-24; and MBLs: NDM, IMP, VIM, SPM and GIM) using multiplex PCR assays, as previously described [17].

Original-spectrum β-lactamases (TEM-type β-lactamases without substitutions at amino acid positions 104, 164 or 238, or SHV-type β-lactamases without substitutions at amino acid positions 146, 238 or 240) were not included. All detected genes were amplified using flanking primers and sequenced. Sequences were compared with publically available databases.

An MDR phenotype among isolates of *P. aeruginosa* was defined as resistance to one or more antimicrobial agents (given in parentheses) from three or more of the following anti-pseudomonal antimicrobial classes: aminoglycosides (amikacin), carbapenems (imipenem or meropenem), cephalosporins (ceftazidime or cefepime), fluoroquinolones (levofloxacin), β-lactam/β-lactamase inhibitor combinations (piperacillin–tazobactam), monobactams (aztreonam), and polymyxins (colistin) [18].

## Results

### Study centers and infection sources of isolates

Twenty-nine centers in the Asia–Pacific region collected Gram-negative isolates between 2015 and 2017: Australia, 7; Japan, 5; South Korea, 3; Malaysia, 2; the Philippines, 5; Taiwan, 4; and Thailand, 3. A total of 7051 Enterobacterales isolates and 2032 *P. aeruginosa* isolates were collected. Enterobacterales isolates were collected from the following sources of infection: urinary tract, 27.5% (n = 1941); lower respiratory tract, 27.1% (n = 1913); skin and skin structure, 21.1% (n = 1485); intra-abdominal, 14.5% (n = 1020) and blood, 9.8% (n = 692). *P. aeruginosa* isolate infection sources were: lower respiratory tract, 50.8% (n = 1032); skin and skin structure, 23.9% (n = 486); urinary tract, 14.0% (n = 285); intra-abdominal, 7.0% (n = 143); blood, 4.1% (n = 84); and other (skeletal), 0.1% (n = 2).

### Resistance in the Asia–Pacific region and by country among all Enterobacterales

**Fig. 1:**
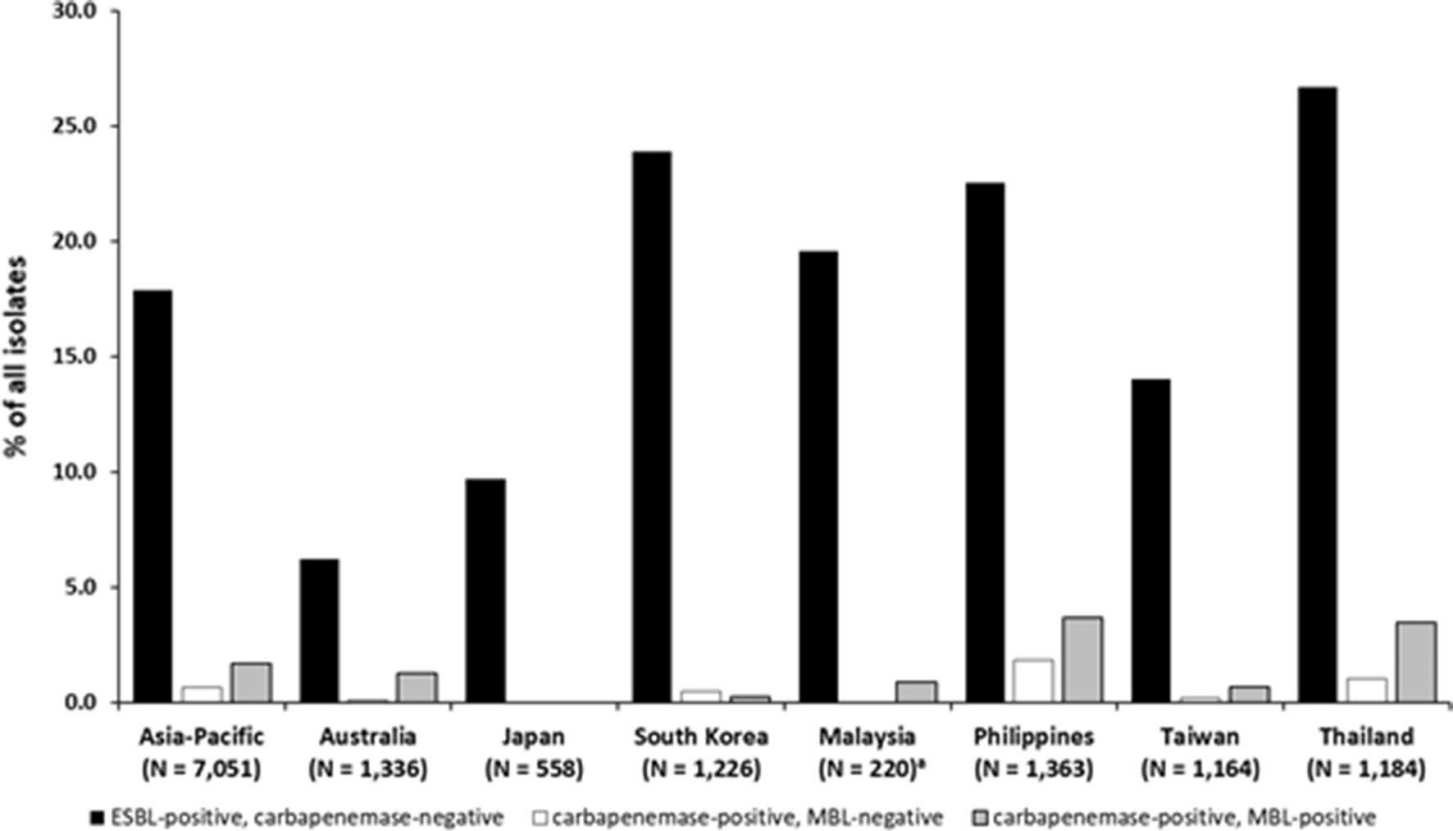
Rates of resistant subsets (ESBL-positive, carbapenemase-negative; carbapenemase-positive, MBL-negative; and carbapenemase-positive, MBL-positive) among Enterobacterales isolates collected in the Asia–Pacific region (INFORM program, 2015–2017). ESBL, extended-spectrum β-lactamase; metallo-β-lactamase. Enterobacterales isolates (N = 7051) comprised: *Citrobacter amalonaticus*, 9; *Citrobacter braakii*, 22; *Citrobacter farmeri*, 8; *Citrobacter freundii*, 311; *Citrobacter koseri*, 231; *Citrobacter murliniae*, 1; *Citrobacter sedlakii*, 2; *Enterobacter asburiae*, 63; *Enterobacter cloacae*, 439; *Enterobacter kobei*, 22; *Enterobacter ludwigii*, 6; *Enterobacter*, non-speciated, 1; *Escherichia coli*, 2218; *Klebsiella aerogenes*, 287; *Klebsiella oxytoca*, 308; *Klebsiella pneumoniae*, 2082; *Klebsiella variicola*, 47; *Morganella morganii*, 164; *Pluralibacter gergoviae*, 6; *Proteus hauseri*, 56; *Proteus mirabilis*, 357; *Proteus penneri*, 7; *Proteus vulgaris*, 151; *Providencia rettgeri*, 61; *Providencia stuartii*, 65; *Raoultella ornithinolytica*, 5; *Raoultella planticola*, 1; *Serratia liquefaciens*, 2; *Serratia marcescens*, 118 and *Serratia*, non-speciated, 1. ^a^Centers in Malaysia collected isolates in 2015 only

Figure 1 shows the rates of resistant subsets among Enterobacterales isolates from the Asia–Pacific region and by country. In the Asia–Pacific region as a whole, 17.9% of the Enterobacterales were ESBL-positive and carbapenemase-negative, 0.7% were carbapenemase-positive and MBL-negative, and 1.7% were carbapenemase-positive and MBL-positive. The country rates of ESBL-positive, carbapenemase-negative Enterobacterales were highest in Thailand (26.7%), South Korea (23.9%) and the Philippines (22.5%), and were lowest in Australia (6.2%) and Japan (9.7%). In each country, the rate of carbapenemase-positive, MBL-negative Enterobacterales was < 2% and the rate of carbapenemase-positive, MBL-positive isolates was < 4%. No carbapenemase-positive isolates (MBL-negative or MBL-positive) were identified among Enterobacterales from Japan, and no Enterobacterales isolates from Malaysia were carbapenemase-positive, MBL-negative.

### Resistance in the Asia–Pacific region and by country among *E. coli* and *K. pneumoniae*

For all *E. coli* and *K. pneumoniae* isolates collected in the Asia–Pacific region, the rate of ESBL-positive, carbapenemase-negative *E. coli* was greater than for *K. pneumoniae* (30.1% and 25.6%, respectively; Table 1). Rates of carbapenemase-positive, MBL-negative and carbapenemase-positive, MBL-positive *K. pneumoniae* in the Asia–Pacific region (1.9% and 2.5%, respectively) were greater than for *E. coli* (0.2% and 0.6%, respectively). By country, the highest rate of ESBL-positive, carbapenemase-negative *E. coli* was collected in Thailand (51.8%) and the highest rates of ESBL-positive, carbapenemase-negative *K. pneumoniae* were collected in South Korea, Malaysia, the Philippines and Thailand (33.5–38.8%). The rates of ESBL-positive, carbapenemase-negative *E. coli* and *K. pneumoniae* were both lowest in Australia (12.2% and 7.6%, respectively). Country rates of carbapenemase-positive, MBL-negative and carbapenemase-positive, MBL-positive *E. coli* were ≤ 0.3%, with the exception of the Philippines (carbapenemase-positive, MBL-positive *E. coli*, 0.9%) and Thailand (0.8% and 2.2%, respectively). The rates of carbapenemase-positive, MBL-negative and carbapenemase-positive, MBL-positive *K. pneumoniae* were highest in the Philippines (5.2% and 4.3%, respectively) and Thailand (2.5% and 7.3%, respectively).

**Table 1:** Rates of resistant subsets among *Escherichia coli* and *Klebsiella pneumoniae* isolates collected in the Asia–Pacific region (INFORM program, 2015–2017)

Organism/region/country	Total isolates	ESBL-positive, carbapenemase-negative		Carbapenemase-positive, MBL-negative		Carbapenemase-positive, MBL-positive	
	N	N	%	N	%	N	%
*Escherichia coli*							
Asia–Pacific	2218	668	30.1	4	0.2	14	0.6
Australia	402	49	12.2	0	0.0	1	0.2
Japan	167	33	19.8	0	0.0	0	0.0
South Korea	449	160	35.6	0	0.0	1	0.2
Malaysia^a^	72	12	16.7	0	0.0	0	0.0
Philippines	431	142	32.9	0	0.0	4	0.9
Taiwan	340	87	25.6	1	0.3	0	0.0
Thailand	357	185	51.8	3	0.8	8	2.2
*Klebsiella pneumoniae*							
Asia–Pacific	2082	532	25.6	39	1.9	52	2.5
Australia	381	29	7.6	1	0.3	6	1.6
Japan	155	18	11.6	0	0.0	0	0.0
South Korea	335	123	36.7	5	1.5	0	0.0
Malaysia^a^	80	31	38.8	0	0.0	1	1.3
Philippines	439	147	33.5	23	5.2	19	4.3
Taiwan	334	58	17.4	1	0.3	0	0.0
Thailand	358	126	35.2	9	2.5	26	7.3

^a^Centers in Malaysia collected isolates in 2015 only

### Resistance in the Asia–Pacific region and by country among *P. aeruginosa*

**Fig. 2:**
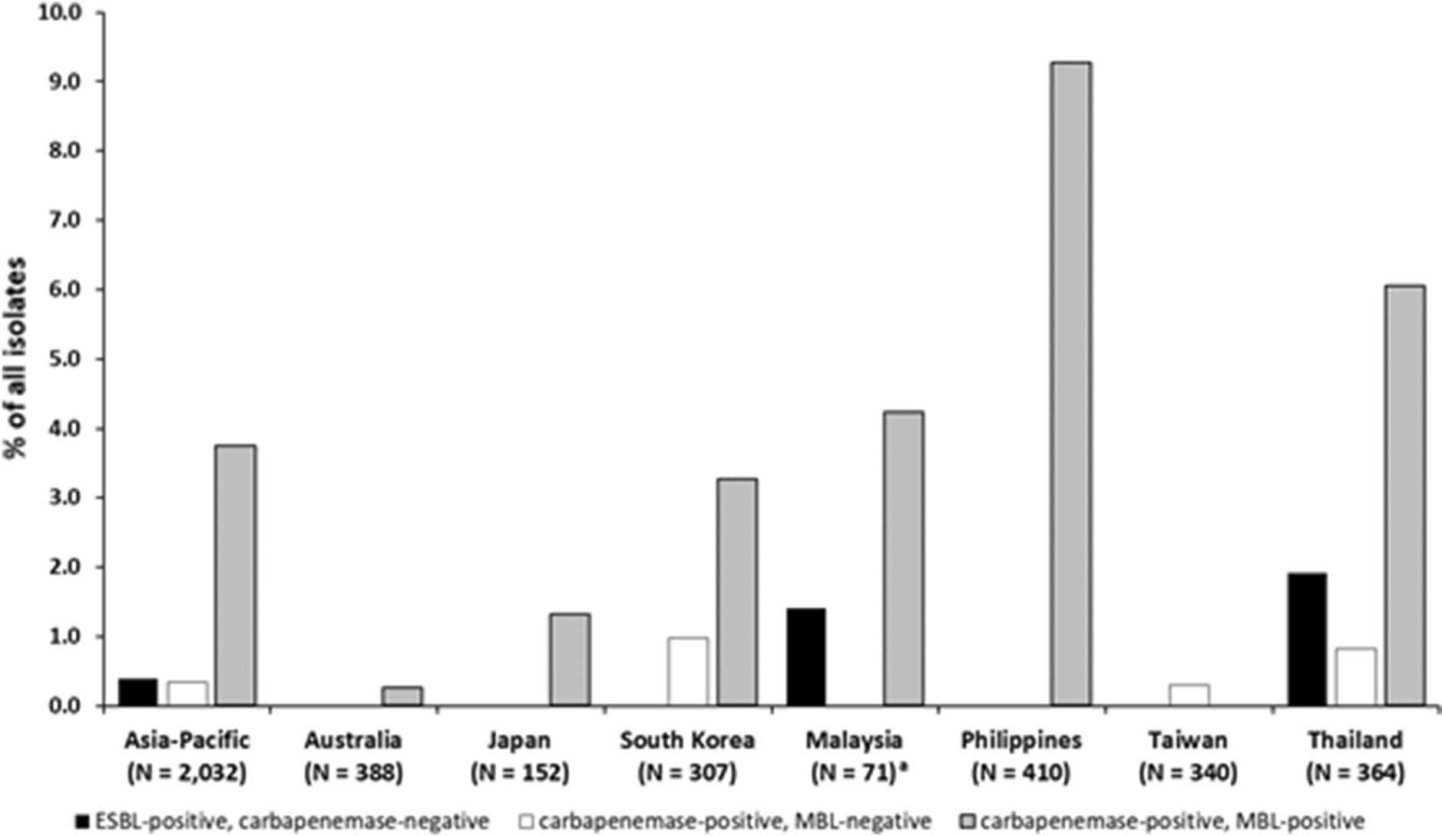
Rates of resistant subsets (ESBL-positive, carbapenemase-negative; carbapenemase-positive, MBL-negative; and carbapenemase-positive, MBL-positive) among *Pseudomonas aeruginosa* isolates collected in the Asia–Pacific region (INFORM program, 2015–2017). ESBL, extended-spectrum β-lactamase; MBL, metallo-β-lactamase. ^a^Centers in Malaysia collected isolates in 2015 only

Figure 2 shows the rates of resistant subsets among *P. aeruginosa* isolates from the Asia–Pacific region and by country. Among all *P. aeruginosa* isolates collected in the Asia–Pacific region, the rate of carbapenemase-positive, MBL-positive isolates (3.7%) was greater than for ESBL-positive, carbapenemase-negative or carbapenemase-positive, MBL-negative isolates (0.4% and 0.3%, respectively). No ESBL-positive, carbapenemase-negative *P. aeruginosa* isolates were collected in Australia, Japan, South Korea, the Philippines, or Taiwan and no carbapenemase-positive, MBL-negative *P. aeruginosa* isolates were collected in Australia, Japan, Malaysia, or the Philippines. The rates of carbapenemase-positive, MBL-positive *P. aeruginosa* were highest in the Philippines (9.3%) and lowest in Taiwan and Australia (0.0% and 0.3%, respectively). The rate of MDR phenotypes among all *P. aeruginosa* isolates in the Asia–Pacific region was 23.4% and the country rates were: Australia, 14.9%; Malaysia, 18.3%; Taiwan, 19.1%; Japan, 21.1%; the Philippines, 24.1%; South Korea, 30.9%; and Thailand, 31.0%.

### In vitro antimicrobial activity of 12 agents in the Asia–Pacific region against all Enterobacterales

The in vitro antimicrobial activities of a panel of agents against the pooled collection of Enterobacterales are presented in Table 2. Among all Enterobacterales isolates, the highest susceptibility rates of 96.8% to 98.1% were observed for amikacin, ceftazidime–avibactam, and meropenem. The activity of ceftazidime alone (MIC_90_, 64 mg/L) was lower than that of ceftazidime–avibactam (MIC_90_, 0.5 mg/L). Susceptibility to colistin was 83.9% and a colistin MIC_90_ value of ≥ 16 mg/L was observed against the Enterobacterales overall; however, colistin was active against *E. coli* and *K. pneumoniae* isolates (MIC_90_, 0.5 and 1 mg/L, respectively). Tigecycline was active against the collection of Enterobacterales (MIC_90_, 1 mg/L).

**Table 2:** In vitro antimicrobial activity of ceftazidime–avibactam and comparators against Enterobacterales, *Escherichia coli,* and *Klebsiella pneumoniae* isolates collected in the Asia–Pacific region (INFORM program, 2015–2017)

Organism/antimicrobial	MIC_50_ (mg/L)	MIC_90_ (mg/L)	MIC range (mg/L)	% S	% R
Enterobacterales (N = 7051)^a^					
Amikacin	2	4	≤ 0.25–≥ 64	96.8	1.6
Amoxicillin–clavulanic acid	8	≥ 64	≤ 0.12–≥ 64	51.3	48.7
Aztreonam	0.12	64	≤ 0.015–≥ 256	69.4	26.6
Cefepime	≤ 0.12	≥ 32	≤ 0.12–≥ 32	75.5	20.3
Ceftazidime	0.25	64	≤ 0.015–≥ 256	69.0	26.7
Ceftazidime–avibactam	0.12	0.5	≤ 0.015–≥ 256	98.1	1.9
Colistin	0.25	≥ 16	≤ 0.06–≥ 16	83.9	16.1
Imipenem	0.25	2	≤ 0.03–≥ 16	85.3	2.3
Levofloxacin	0.12	≥ 16	≤ 0.004–≥ 16	66.2	27.8
Meropenem	0.03	0.12	≤ 0.004–≥ 16	97.7	1.6
Piperacillin–tazobactam	2	64	≤ 0.25–≥ 256	82.5	13.4
Tigecycline^b^	0.5	1	≤ 0.015–≥ 16	N/A	N/A
Enterobacterales, ESBL-positive, carbapenemase-negative (N = 1259)					
Amikacin	2	8	≤ 0.25–≥ 64	91.0	3.7
Amoxicillin–clavulanic acid	16	32	1–≥ 64	26.7	73.3
Aztreonam	64	≥ 256	0.5–≥ 256	0.5	91.2
Cefepime	≥ 32	≥ 32	≤ 0.12–≥ 32	3.2	85.5
Ceftazidime	32	≥ 256	0.25–≥ 256	5.9	79.7
Ceftazidime–avibactam	0.25	0.5	≤ 0.015–≥ 256	99.9	0.1
Colistin	0.25	1	0.12–≥ 16	96.6	3.4
Imipenem	0.25	0.5	0.06–≥ 16	98.1	0.6
Levofloxacin	≥ 16	≥ 16	0.03–≥ 16	18.0	71.7
Meropenem	0.03	0.12	0.008–8	98.6	0.0
Piperacillin–tazobactam	8	≥ 256	≤ 0.25–≥ 256	62.0	26.6
Tigecycline^b^	0.25	1	≤ 0.015–≥ 16	N/A	N/A
Enterobacterales, carbapenemase-positive, MBL-negative (N = 46)					
Amikacin	2	8	≤ 0.25–≥ 64	93.5	2.2
Amoxicillin–clavulanic acid	≥ 64	≥ 64	≥ 64–≥ 64	0.0	100
Aztreonam	≥ 256	≥ 256	8–≥ 256	0.0	100
Cefepime	≥ 32	≥ 32	≤ 0.12–≥ 32	4.3	91.3
Ceftazidime	128	≥ 256	8–≥ 256	0.0	100
Ceftazidime–avibactam	1	2	0.12–4	100	0.0
Colistin	0.25	≥ 16	0.12–≥ 16	87.0	13.0
Imipenem	8	≥ 16	0.5–≥ 16	23.9	67.4
Levofloxacin	≥ 16	≥ 16	0.06–≥ 16	10.9	82.6
Meropenem	≥ 16	≥ 16	0.5–≥ 16	26.1	58.7
Piperacillin–tazobactam	≥ 256	≥ 256	128–≥ 256	0.0	100
Tigecycline^b^	0.5	1	0.12–≥ 16	N/A	N/A
Enterobacterales, carbapenemase-positive, MBL-positive (N = 121)					
Amikacin	4	≥ 64	0.5–≥ 64	75.2	16.5
Amoxicillin–clavulanic acid	≥ 64	≥ 64	16–≥ 64	0.0	100
Aztreonam	128	≥ 256	≤ 0.015–≥ 256	12.4	81.8
Cefepime	≥ 32	≥ 32	2–≥ 32	0.0	94.2
Ceftazidime	≥ 256	≥ 256	32–≥ 256	0.0	100
Ceftazidime–avibactam	≥ 256	≥ 256	32–≥ 256	0.0	100
Colistin	0.5	1	≤ 0.06–≥ 16	93.4	6.6
Imipenem	≥ 16	≥ 16	1–≥ 16	13.2	77.7
Levofloxacin	≥ 16	≥ 16	0.06–≥ 16	11.6	81.8
Meropenem	≥ 16	≥ 16	0.5–≥ 16	9.9	71.1
Piperacillin–tazobactam	≥ 256	≥ 256	1–≥ 256	7.4	90.9
Tigecycline^b^	0.5	4	0.12–8	N/A	N/A
*E. coli* (N = 2218)					
Amikacin	2	8	≤ 0.25–≥ 64	97.1	0.6
Amoxicillin–clavulanic acid	8	32	≤ 0.12–≥ 64	55.7	44.3
Aztreonam	0.12	64	≤ 0.015–≥ 256	62.4	32
Cefepime	≤ 0.12	≥ 32	≤ 0.12–≥ 32	67.6	27.6
Ceftazidime	0.25	32	≤ 0.015–≥ 256	64.0	28.4
Ceftazidime–avibactam	0.12	0.25	≤ 0.015–≥ 256	99.4	0.6
Colistin	0.25	0.5	≤ 0.06–≥ 16	99.3	0.7
Imipenem	0.12	0.25	≤ 0.03–≥ 16	99.2	0.7
Levofloxacin	0.5	≥ 16	≤ 0.004–≥ 16	50.6	45.4
Meropenem	0.03	0.06	0.008–≥ 16	99.1	0.5
Piperacillin–tazobactam	2	16	≤ 0.25–≥ 256	89.0	7.3
Tigecycline^b^	0.25	0.5	0.03–≥ 16	97.7	2.3
*E. coli*, ESBL-positive, carbapenemase-negative (N = 668)					
Amikacin	4	8	0.5–≥ 64	92.8	1.2
Amoxicillin–clavulanic acid	16	32	1–≥ 64	36.4	63.6
Aztreonam	32	128	0.5–≥ 256	0.1	88.9
Cefepime	≥ 32	≥ 32	≤ 0.12–≥ 32	1.6	86.8
Ceftazidime	16	64	0.25–≥ 256	8.4	70.1
Ceftazidime–avibactam	0.12	0.25	≤ 0.015–4	100	0.0
Colistin	0.25	0.5	0.12–8	98.4	1.6
Imipenem	0.12	0.25	0.06–≥ 16	99.7	0.3
Levofloxacin	≥ 16	≥ 16	0.03–≥ 16	14.2	81.9
Meropenem	0.03	0.06	0.008–8	99.6	0.0
Piperacillin–tazobactam	4	32	≤ 0.25–≥ 256	82.2	10.0
Tigecycline^b^	0.25	0.5	0.03–≥ 16	96.9	3.1
*E. coli*, carbapenemase-positive, MBL-positive (N = 14)					
Amikacin	4	≥ 64	2–≥ 64	71.4	21.4
Amoxy/clav	≥ 64	≥ 64	32–≥ 64	0.0	100
Aztreonam	≥ 256	≥ 256	0.12–≥ 256	7.1	92.9
Cefepime	≥ 32	≥ 32	≥ 32–≥ 32	0.0	100
Ceftazidime	≥ 256	≥ 256	128–≥ 256	0.0	100
Ceftazidime–avibactam	≥ 256	≥ 256	64–≥ 256	0.0	100
Colistin	0.25	1	0.12–1	100	0.0
Imipenem	≥ 16	≥ 16	2–≥ 16	7.1	92.9
Levofloxacin	≥ 16	≥ 16	1–≥ 16	0.0	92.9
Meropenem	≥ 16	≥ 16	4–≥ 16	0.0	78.6
Pip/taz	≥ 256	≥ 256	64–≥ 256	0.0	100
Tigecycline^b^	0.25	0.5	0.12–2	92.9	7.1
*K. pneumoniae* (N = 2082)					
Amikacin	1	4	≤ 0.25–≥ 64	95.6	2.5
Amoxicillin–clavulanic acid	4	≥ 64	≤ 0.12–≥ 64	64.0	36.0
Aztreonam	0.12	≥ 256	≤ 0.015–≥ 256	66.1	31.3
Cefepime	≤ 0.12	≥ 32	≤ 0.12–≥ 32	69.7	26.7
Ceftazidime	0.25	≥ 256	≤ 0.015–≥ 256	65.0	32.4
Ceftazidime–avibactam	0.12	0.5	≤ 0.015–≥ 256	97.5	2.5
Colistin	0.25	1	≤ 0.06–≥ 16	98.1	1.9
Imipenem	0.25	1	≤ 0.03–≥ 16	95.5	3.8
Levofloxacin	0.12	≥ 16	0.008–≥ 16	65.4	25.9
Meropenem	0.06	0.12	0.008–≥ 16	95.4	3.3
Piperacillin–tazobactam	4	≥ 256	≤ 0.25–≥ 256	73.0	21.0
Tigecycline^b^	0.5	1	≤ 0.015–≥ 16	N/A	N/A
*K. pneumoniae*, ESBL-positive, carbapenemase-negative (N = 532)					
Amikacin	2	16	≤ 0.25–≥ 64	89.1	6.2
Amoxicillin–clavulanic acid	16	≥ 64	2–≥ 64	11.8	88.2
Aztreonam	128	≥ 256	0.5–≥ 256	0.6	95.1
Cefepime	≥ 32	≥ 32	≤ 0.12–≥ 32	3.4	86.1
Ceftazidime	64	≥ 256	0.25–≥ 256	2.4	91.9
Ceftazidime–avibactam	0.25	1	≤ 0.015–≥ 256	99.8	0.2
Colistin	0.25	1	0.12–≥ 16	96.8	3.2
Imipenem	0.25	1	0.06–≥ 16	98.7	0.8
Levofloxacin	8	≥ 16	0.03–≥ 16	20.3	62.8
Meropenem	0.06	0.12	0.015–8	97.6	0.0
Piperacillin–tazobactam	16	≥ 256	1–≥ 256	35.5	48.5
Tigecycline^b^	0.5	2	≤ 0.015–≥ 16	N/A	N/A
*K. pneumoniae*, carbapenemase-positive, MBL-negative (N = 39)					
Amikacin	2	4	≤ 0.25–≥ 64	94.9	2.6
Amoxicillin–clavulanic acid	≥ 64	≥ 64	≥ 64–≥ 64	0.0	100
Aztreonam	≥ 256	≥ 256	8–≥ 256	0.0	100
Cefepime	≥ 32	≥ 32	≤ 0.12–≥ 32	5.1	92.3
Ceftazidime	128	≥ 256	32–≥ 256	0.0	100
Ceftazidime–avibactam	1	2	0.12–4	100	0.0
Colistin	0.25	≥ 16	0.25–≥ 16	87.2	12.8
Imipenem	8	≥ 16	0.5–≥ 16	20.5	69.2
Levofloxacin	≥ 16	≥ 16	0.06–≥ 16	5.1	87.2
Meropenem	≥ 16	≥ 16	0.5–≥ 16	23.1	61.5
Piperacillin–tazobactam	≥ 256	≥ 256	128–≥ 256	0.0	100
Tigecycline^b^	0.5	2	0.25–≥ 16	N/A	N/A
*K. pneumoniae*, carbapenemase-positive, MBL-positive (N = 52)					
Amikacin	4	≥ 64	0.5–≥ 64	67.3	17.3
Amoxicillin–clavulanic acid	≥ 64	≥ 64	16–≥ 64	0.0	100
Aztreonam	128	≥ 256	0.06–≥ 256	3.8	94.2
Cefepime	≥ 32	≥ 32	4–≥ 32	0.0	94.2
Ceftazidime	≥ 256	≥ 256	128–≥ 256	0.0	100
Ceftazidime– avibactam	≥ 256	≥ 256	32–≥ 256	0.0	100
Colistin	0.5	1	0.12–≥ 16	94.2	5.8
Imipenem	≥ 16	≥ 16	1–≥ 16	5.8	88.5
Levofloxacin	≥ 16	≥ 16	0.25–≥ 16	3.8	86.5
Meropenem	≥ 16	≥ 16	2–≥ 16	1.9	84.6
Piperacillin–tazobactam	≥ 256	≥ 256	16–≥ 256	0.0	96.2
Tigecycline^b^	1	4	0.12–8	N/A	N/A

^a^Enterobacterales isolates (N = 7051) comprised: *Citrobacter amalonaticus*, 9; *Citrobacter braakii*, 22; *Citrobacter farmeri*, 8; *Citrobacter freundii*, 311; *Citrobacter koseri*, 231; *Citrobacter murliniae*, 1; *Citrobacter sedlakii*, 2; *Enterobacter asburiae*, 63; *Enterobacter cloacae*, 439; *Enterobacter kobei*, 22; *Enterobacter ludwigii*, 6; *Enterobacter*, non-speciated, 1; *Escherichia coli*, 2218; *Klebsiella aerogenes*, 287; *Klebsiella oxytoca*, 308; *Klebsiella pneumoniae*, 2082; *Klebsiella variicola*, 47; *Morganella morganii*, 164; *Pluralibacter gergoviae*, 6; *Proteus hauseri*, 56; *Proteus mirabilis*, 357; *Proteus penneri*, 7; *Proteus vulgaris*, 151; *Providencia rettgeri*, 61; *Providencia stuartii*, 65; *Raoultella ornithinolytica*, 5; *Raoultella planticola*, 1; *Serratia liquefaciens*, 2; *Serratia marcescens*, 118 and *Serratia*, non-speciated, 1

^b^EUCAST breakpoints for tigecycline are only available with *E. coli*

Among ESBL-positive, carbapenemase-negative Enterobacterales isolates, susceptibility to amikacin, ceftazidime–avibactam, colistin, imipenem and meropenem was ≥ 91% (Table 2). The MIC_90_ value for tigecycline was 1 mg/L. Fewer than 6% of ESBL-positive, carbapenemase-negative isolates were susceptible to aztreonam, cefepime or ceftazidime. Among the carbapenemase-positive, MBL-negative subset, susceptibility was highest to ceftazidime–avibactam (100%), amikacin (93.5%), and colistin (87.0%). For the majority of agents with EUCAST breakpoints, susceptibility was reduced among carbapenemase-positive, MBL-positive isolates, compared with the Enterobacterales overall. Colistin was the only agent to which susceptibility was maintained for the carbapenemase-positive, MBL-positive subset (93.4%). No carbapenemase-positive, MBL-positive isolates were susceptible to ceftazidime–avibactam, compared with 100% of carbapenemase-positive, MBL-negative isolates. No carbapenemase-positive, MBL-negative or carbapenemase-positive, MBL-positive isolates were susceptible to amoxicillin–clavulanic acid or ceftazidime.

### In vitro antimicrobial activity of 12 agents in the Asia–Pacific region against *E. coli* and *K. pneumoniae*

More than 95% of all *E. coli* and *K. pneumoniae* isolates were susceptible to amikacin, ceftazidime–avibactam, colistin, imipenem and meropenem (Table 2). Among *E. coli* isolates, 97.7% were susceptible to tigecycline and a tigecycline MIC_90_ value of 0.5 mg/L was observed. No EUCAST breakpoints are available for tigecycline against *K. pneumoniae*. Susceptibility rates to amikacin, ceftazidime–avibactam, colistin, imipenem and meropenem were > 89% among ESBL-positive, carbapenemase-negative *E. coli* and *K. pneumoniae* isolates. Among ESBL-positive, carbapenemase-negative *E. coli* isolates, 96.9% were susceptible to tigecycline.

Fewer carbapenemase-positive, MBL-negative and carbapenemase-positive, MBL-positive *E. coli* isolates (N = 4 and N = 14, respectively) were collected than for those of *K. pneumoniae* (N = 39 and N = 52, respectively; Table 2). Data for the carbapenemase-positive, MBL-negative subset of *E. coli* are not presented in Table 2 because of the small isolate numbers. Against this subset, the lowest MIC ranges were observed for colistin and tigecycline (both agents, 0.12–0.5 mg/L), followed by ceftazidime–avibactam (0.25–2 mg/L), whereas amoxicillin–clavulanic acid, aztreonam, ceftazidime, and piperacillin–tazobactam were not active. For the carbapenemase-positive, MBL-positive subset of *E. coli* and the carbapenemase-positive, MBL-negative and carbapenemase-positive, MBL-positive subsets of *K. pneumoniae*, the susceptibility rates to the majority of agents were < 24%, with the exception of amikacin (67.3–94.9%) and colistin (87.2–100%; Table 2). Susceptibility to tigecycline among carbapenemase-positive, MBL-positive *E. coli* isolates was 92.9%. All carbapenemase-positive, MBL-negative *K. pneumoniae* isolates were susceptible to ceftazidime–avibactam; however, no carbapenemase-positive, MBL-positive *E. coli* or *K. pneumoniae* isolates were susceptible to ceftazidime–avibactam.

### In vitro antimicrobial activity of 12 agents in the Asia–Pacific region against *P. aeruginosa*

The highest rate of susceptibility among *P. aeruginosa* isolates was to colistin (99.7%), followed by ceftazidime–avibactam and amikacin (92.7% and 92.3%, respectively; Table 3). The activity of ceftazidime alone (MIC_90_, 64 mg/L) was less potent than that of ceftazidime–avibactam (MIC_90_, 8 mg/L). Among the resistant subsets of *P. aeruginosa*, eight isolates were identified as ESBL-positive, carbapenemase-negative and seven as carbapenemase-positive, MBL-negative. These data are not presented in Table 3 because of the small isolate numbers; however, the lowest MIC range was observed for colistin (0.5–2 mg/L against both subsets). Susceptibility to the majority of agents was reduced among the carbapenemase-positive, MBL-positive subset, when compared with the susceptibility among all *P. aeruginosa* isolates, with the exception of colistin (100% susceptible; Table 3). Amoxicillin–clavulanic acid (MIC_90_, ≥ 64 mg/L) and tigecycline (MIC_90_, ≥ 16 mg/L) were inactive against all MDR, or carbapenemase-positive, MBL-positive *P. aeruginosa* isolates.

**Table 3:** In vitro antimicrobial activity of ceftazidime–avibactam and comparators against *Pseudomonas aeruginosa* isolates collected in the Asia–Pacific region (INFORM program, 2015–2017)

Organism/antimicrobial	MIC_50_ (mg/L)	MIC_90_ (mg/L)	MIC range (mg/L)	% S	% R
*Pseudomonas aeruginosa* (N = 2032)					
Amikacin	4	8	≤ 0.25–≥ 64	92.3	5.2
Amoxicillin–clavulanic acid^a^	≥ 64	≥ 64	0.5–≥ 64	N/A	N/A
Aztreonam	8	32	≤ 0.015–≥ 256	80.6	19.4
Cefepime	4	≥ 32	≤ 0.12–≥ 32	81.9	18.1
Ceftazidime	2	64	0.06–≥ 256	78.4	21.6
Ceftazidime–avibactam	2	8	≤ 0.015–≥ 256	92.7	7.3
Colistin	1	2	≤ 0.06–8	99.7	0.3
Imipenem	2	≥ 16	0.12–≥ 16	80.0	20.0
Levofloxacin	0.5	≥ 16	0.008–≥ 16	68.5	31.5
Meropenem	0.5	≥ 16	0.008–≥ 16	80.3	10.8
Piperacillin–tazobactam	8	≥ 256	≤ 0.25–≥ 256	73.7	26.3
Tigecycline^a^	8	≥ 16	≤ 0.015–≥ 16	N/A	N/A
*P. aeruginosa*, MDR (N = 475)					
Amikacin	4	≥ 64	≤ 0.25–≥ 64	71.6	21.5
Amoxicillin–clavulanic acid^a^	≥ 64	≥ 64	32–≥ 64	N/A	N/A
Aztreonam	32	128	0.25–≥ 256	25.9	74.1
Cefepime	16	≥ 32	1–≥ 32	26.3	73.7
Ceftazidime	64	≥ 256	0.5–≥ 256	20.2	79.8
Ceftazidime–avibactam	8	128	0.25–≥ 256	68.6	31.4
Colistin	1	2	0.12–2	100	0.0
Imipenem	≥ 16	≥ 16	0.25–≥ 16	41.1	58.9
Levofloxacin	8	≥ 16	0.06–≥ 16	25.1	74.9
Meropenem	8	≥ 16	0.12–≥ 16	38.3	43.8
Piperacillin–tazobactam	128	≥ 256	≤ 0.25–≥ 256	5.1	94.9
Tigecycline^a^	≥ 16	≥ 16	0.03–≥ 16	N/A	N/A
*P. aeruginosa*, carbapenemase-positive, MBL-positive (N = 76)					
Amikacin	32	≥ 64	2–≥ 64	10.5	76.3
Amoxicillin–clavulanic acid^a^	≥ 64	≥ 64	≥ 64–≥ 64	N/A	N/A
Aztreonam	16	≥ 256	0.25–≥ 256	52.6	47.4
Cefepime	≥ 32	≥ 32	16–≥ 32	0.0	100
Ceftazidime	128	≥ 256	16–≥ 256	0.0	100
Ceftazidime–avibactam	128	≥ 256	2–≥ 256	3.9	96.1
Colistin	1	1	0.5–2	100	0.0
Imipenem	≥ 16	≥ 16	4–≥ 16	1.3	98.7
Levofloxacin	≥ 16	≥ 16	0.5–≥ 16	3.9	96.1
Meropenem	≥ 16	≥ 16	8–≥ 16	0.0	96.1
Piperacillin–tazobactam	128	≥ 256	8–≥ 256	6.6	93.4
Tigecycline^a^	≥ 16	≥ 16	4–≥ 16	N/A	N/A

^a^EUCAST breakpoints for amoxicillin–clavulanic acid and tigecycline are not available with *P. aeruginosa*

## Discussion

This study reports the in vitro antimicrobial susceptibilities and the rates of resistant subsets for clinical isolates of Enterobacterales and *P. aeruginosa* from the Asia–Pacific region. For the pooled collection of Enterobacterales, susceptibility was highest to amikacin, ceftazidime–avibactam and meropenem, and among *P. aeruginosa* was highest to amikacin, ceftazidime–avibactam and colistin. Enterobacterales isolates that were ESBL-positive, carbapenemase-negative were more common than carbapenemase-positive, MBL-negative or carbapenemase-positive, MBL-positive isolates. MDR isolates of *P. aeruginosa* were more frequently observed than the other resistant subsets.

This study is an update to the 2012–2015 INFORM study, which reported similar susceptibility rates to amikacin, ceftazidime–avibactam, colistin and meropenem among a pooled collection of Enterobacteriaceae [11]. Any comparisons should be treated with caution, however, as the isolates collected in 2015 were included in both studies and the 2012–2015 INFORM study also included isolates from China and Hong Kong. Yet, the findings of these studies together would indicate a level of stability in antimicrobial susceptibility among the Enterobacterales in the Asia–Pacific region.

In the current study, susceptibility to colistin and its antimicrobial activity was lower for the pooled collection of Enterobacterales, when compared with *E. coli* and *K. pneumoniae* isolates individually. This reduced susceptibility to colistin among the pooled group of Enterobacterales is likely to be due to the presence of intrinsically colistin-resistant species, such as *P. mirabilis* and *Serratia marcescens* [19].

Among the resistant subsets presented in this study, > 98% of ESBL-positive, carbapenemase-negative Enterobacterales isolates remained susceptible to amikacin, ceftazidime–avibactam, colistin, imipenem and meropenem. The range of antimicrobial susceptibilities to these agents was similar to the 2012–2015 INFORM study for the isolates of Enterobacteriaceae molecularly-characterized as ESBL-positive (95.1–99.6%) [11]. In the present study, susceptibility to colistin, imipenem and meropenem was reduced among the carbapenemase-positive, MBL-negative subset of Enterobacterales, compared with the ESBL-positive, carbapenemase-negative subset; however, susceptibility to amikacin and ceftazidime–avibactam among this subset remained > 93%. Similar trends were observed among *K. pneumoniae* isolates, although there were relatively low isolate numbers in the resistant subsets for this species. Studies of antimicrobial resistance in South Korea and Taiwan, on isolates collected between 2012 and 2017, have reported the rates of amikacin resistance to be < 10% among *E. coli* and *K. pneumoniae* isolates [20–22], and among carbapenem-nonsusceptible *E. coli* isolates (Taiwan only) [23].

In the current study, the group of carbapenemase-positive, MBL-positive Enterobacterales remained susceptible to colistin (93.4%) and similar results were seen for these resistant subsets of *K. pneumoniae* (94.2%) and *E. coli* (100%) isolates. Furthermore, nearly 100% of *P. aeruginosa* isolates, including all MDR or carbapenemase-positive, MBL-positive isolates, were susceptible to colistin. Colistin has been described in the literature as a ‘last-resort’ antimicrobial agent due to its clinical efficacy against antimicrobial-resistant isolates of Enterobacterales and *P. aeruginosa* [19]. However, resistance to colistin, along with the carbapenems, has been emerging in Southeast Asia, leading to increased hospital mortality rates in the case of the carbapenems [5]. A clinical trial involving patients with infections predominantly caused by carbapenem-resistant *K. pneumoniae* found that ceftazidime–avibactam was superior to colistin and may be an alternative treatment option [24]. Avibactam is inactive against MBL-positive isolates, therefore it is presumed that carbapenem resistance among *K. pneumoniae* from these clinical studies was mediated by the serine carbapenemases, such as KPC and OXA-48 [8, 25], which are susceptible to ceftazidime–avibactam.

For *P. aeruginosa* isolates in the current study, the MDR subset accounted for 23.4%. Lower rates of MDR *P. aeruginosa* were recently reported in the literature for the Asia–Pacific region (15.0% [2013–2016] and 14.8% [2012–2015]) [11, 26]. Each study used the MDR definition proposed by Magiorakos et al. [18]; however, variations in the participating countries and time periods for each study may account for the differences in the overall regional rates.

One of the limitations of this antimicrobial surveillance study is that a predefined number of isolates were collected at each center, so the findings cannot describe the epidemiology of resistance. Additionally, the lower numbers of isolates collected from Japan and Malaysia than elsewhere may have been insufficient to allow the detection of resistant subsets, resulting in relatively low resistant rates being reported for these countries. Furthermore, this study did not include data from China and India where antimicrobial resistance is prevalent, exemplified by the discovery of the first colistin-resistant Enterobacteriaceae isolate [27] and the first *bla*_NDM-1_ gene in *K. pneumoniae* [28] in these two countries, respectively. Nevertheless, reporting the local antimicrobial activities of contemporary agents in this region, through programs such as INFORM, is a key to guide physicians toward appropriate use of antimicrobials.

## Conclusions

In the Asia–Pacific region, ESBL-positive, carbapenemase-negative Enterobacterales (17.9%) were identified more frequently than carbapenemase-positive, MBL-negative (0.7%) or carbapenemase-positive, MBL-positive isolates (1.7%). The rates of ceftazidime–avibactam, meropenem and amikacin susceptibility were highest (> 90%) among all Enterobacterales, including ESBL-positive, carbapenemase-negative, and carbapenemase-positive, MBL-negative isolates. Among the carbapenemase-positive, MBL-positive Enterobacterales, the rate of meropenem susceptibility (9.9%) was notably reduced, and no isolates in this subset were susceptible to ceftazidime–avibactam. For *P. aeruginosa*, carbapenemase-positive, MBL-positive isolates (3.7%) were more common than ESBL-positive, carbapenemase-negative (0.4%), or carbapenemase-positive, MBL-negative isolates (0.3%).

Among all *P. aeruginosa* isolates, including the resistant subsets, the rate of colistin susceptibility (> 99%) was higher than rates for ceftazidime–avibactam and amikacin, particularly among carbapenemase-positive, MBL-positive isolates (3.9% and 10.5%, respectively). Data from this study have shown the variability in antimicrobial activity depending on the β-lactamase profile of isolates, and multicenter surveillance of antimicrobial resistance remains essential for public health and clinical management of bacterial infections.

## Data Availability

Data from the study can be accessed at https://atlas-surveillance.com. The datasets used and/or analyzed during the current study of isolates collected in the Asia–Pacific region, 2015 to 2017, are available from the corresponding author on reasonable request.

## Abbreviations

CLSI: Clinical Laboratory Standards Institute
ESBL: Extended-spectrum β-lactamase
EUCAST: European Committee on Antimicrobial Susceptibility Testing
INFORM: International Network for Optimal Resistance Monitoring
KPC: *Klebsiella pneumoniae* carbapenemase
MBL: Metallo-β-lactamase
MDR: Multidrug-resistant
MIC: Minimum inhibitory concentration (mg/L)
MIC_50_: MIC required to inhibit growth of 50% of isolates (mg/L)
MIC_90_: MIC required to inhibit growth of 90% of isolates (mg/L)
N/A: Not available
PCR: Polymerase chain reaction
R: Resistant
S: Susceptible (standard dosing)
